# Evaluation of the Anti-Inflammatory Effects of Novel Fatty Acid-Binding Protein 4 Inhibitors in Microglia

**DOI:** 10.1007/s11481-025-10191-9

**Published:** 2025-04-16

**Authors:** Yi Ling Low, Ethan Kreutzer, Indu R. Chandrashekaran, Luke A. Adams, Jason Pun, Bradley C. Doak, Yijun Pan, Jennifer L. Short, Martin J. Scanlon, Joseph A. Nicolazzo

**Affiliations:** 1 Drug Delivery, Disposition and Dynamics, Monash Institute of Pharmaceutical Sciences, Monash University, Parkville, VIC 3052 Australia; 2 Medicinal Chemistry, Monash Institute of Pharmaceutical Sciences, Monash University, Parkville, VIC 3052 Australia; 3 ARC Centre for Fragment-Based Design, Monash Institute of Pharmaceutical Sciences, Monash University, Parkville, VIC 3052 Australia; 4 Monash Fragment Platform, Monash Institute of Pharmaceutical Sciences, Monash University, Parkville, VIC 3052 Australia; 5 Florey Institute of Neuroscience and Mental Health, University of Melbourne, Melbourne, VIC 3052 Australia; 6 Monash Centre for Advanced mRNA Medicines Manufacturing and Workforce Training, Monash University, Clayton, VIC Australia; 7 Centre for Drug Candidate Optimisation, Monash Institute of Pharmaceutical Sciences, Monash University, Parkville, VIC 3052 Australia

**Keywords:** Fatty acid-binding protein 4, Microglia, Reactive oxygen species, Tumour necrosis factor alpha, Neuroinflammation

## Abstract

Fatty acid-binding protein 4 (FABP4) is a key lipid binding protein expressed in microglia, which has been demonstrated to play a critical role in microglial-mediated neuroinflammation, a component of many neurodegenerative diseases. Compounds able to inhibit the function of FABP4 have shown promise in reducing microglial-mediated neuroinflammation, however, their physicochemical properties would prevent their ability to be easily formulated and traverse the blood–brain barrier (BBB) in order to access microglial FABP4. To this end, this study assessed the ability of a series of FABP4 inhibitors, with more desirable physicochemical properties, to attenuate microglial inflammation in an in vitro setting. Four inhibitors with varying affinity to FABP4, as measured by isothermal titration calorimetry (MFP-0011462, MFP-0012314, MFP-0012318, and MFP-0012328), were assessed for their ability to induce toxicity and attenuate reactive oxygen species (ROS) generation and tumour necrosis factor-α (TNF-α) release from lipopolysaccharide (LPS)-activated BV-2 microglia. All FABP4 inhibitors were determined to be soluble in the aqueous buffers at the highest concentration used in the assays (100 µM). Isothermal titration calorimetry demonstrated that the compounds had varying affinities for FABP4 (K_D_ values of 316 nM to > 100 µM). The ability of FABP4 inhibitors to reduce LPS-mediated ROS production aligned with their K_D_ for FABP4, with the most effective inhibitor (MFP-0012328) also able to reduce TNF-α production (by RT-qPCR) and TNF-α release from LPS-activated BV-2 cells by 17% and 25%, respectively. These studies have demonstrated that a series of FABP4 inhibitors with more appropriate physicochemical properties for BBB penetration are able to reduce microglial-mediated inflammation, which may be of benefit in diseases where overactivation of microglia leads to neurodegeneration.

**Supplementary Information:**

The online version contains supplementary material available at 10.1007/s11481-025-10191-9.

## Introduction

Neuroinflammation is defined as an inflammatory process of the central nervous system (CNS), and it is a common pathology associated with various neurodegenerative diseases, such as Alzheimer’s disease (AD), Parkinson’s disease (PD), and motor neuron disease (MND) (Gao et al. 2023). Neuroinflammation is characterised by the excessive release of proinflammatory cytokines from microglia, the main immune cell of the CNS. A continued elevation in the concentrations of proinflammatory molecules such as interleukin-1β (IL-1β), tumour necrosis factor-α (TNF-α), reactive oxygen species (ROS), and nitric oxide (NO) can lead to severe reductions in neuronal number and function (Araujo Boleti et al. 2020). Given that microglia are heavily involved in the neuroinflammatory process, there is growing interest in targeting microglial activation as a therapeutic approach to reduce neurodegenerative processes associated with diseases such as AD, PD, and MND (Subhramanyam et al. 2019).

Recent studies have reported on the involvement of a fatty acid-binding protein (FABP) isoform in the activation process in both macrophages and microglia (Hui et al. 2010; Kagawa et al. 2023). FABPs are a family of non-catalytic chaperones that are capable of binding to a variety of fatty acids and other hydrophobic ligands and they regulate a number of biological processes in cells with a high fatty acid turnover (Zimmerman et al. 2001). One FABP isoform, adipose FABP, otherwise known as FABP4, is expressed largely in adipocytes and plays an important role in various metabolic and inflammatory pathways. FABP4 promotes lipolysis and therefore contributes to the accumulation of lipids and the development of harmful secondary conditions such as type 2 diabetes and atherosclerosis (Furuhashi and Hotamisligil 2008; Furuhashi et al. 2007). In addition to its roles in adipocytes, FABP4 is expressed in peripheral macrophages, where it is known to promote a pro-inflammatory response (Furuhashi et al. 2007). In peripheral inflammatory disorders, such as obesity, cardiovascular disease, atherosclerosis and rheumatoid arthritis, the expression of FABP4 in macrophages is increased, and pharmacological inhibition through FABP4 inhibitors, such as BMS309403, reduces negative outcomes in animal models of these peripheral diseases (Furuhashi et al. 2007; Guo et al. 2022; Lan et al. 2011). While most FABP4 research has focused on adipocytes and peripheral macrophages, we have more recently confirmed that FABP4 is expressed in BV-2 cells, an immortalised mouse microglial cell line, at both the mRNA and protein level and in primary mouse microglia at the mRNA level (Low et al. 2021). As in the periphery, FABP4 plays a significant role in mediating the release of inflammatory mediators from microglia, which has been suggested to occur through the role of FABP4 in inhibiting the anti-oxidative mediator uncoupling protein 2 (Duffy et al. 2017). Brain cortical expression of FABP4 has been shown to be increased in a HIV-induced model of neuroinflammation, which was mediated through nuclear factor kappa B (NF-κB), and in this model, genetic inhibition of microglial FABP4 attenuated neuroinflammation (Zhou et al. 2022). Similarly, it was demonstrated that genetic and chemical inhibition of FABP4 (through BMS309403) significantly reduced the lipopolysaccharide (LPS)-induced release of proinflammatory molecules from BV-2 cells and in primary mouse microglia (Kagawa et al. 2023). These studies suggest that inhibiting the function of FABP4 may be a novel therapeutic approach to reduce microglial-mediated neuroinflammation.

Studies have reported on various FABP4 inhibitors being synthesised for the treatment of diabetes and atherosclerosis, including derivatives of niacin, quinoxaline, aryl-quinoline, bicyclic pyridine, urea, aromatic compounds, and other heterocyclic compounds (Floresta et al. 2017). These inhibitors have been widely studied for their protein-drug interactions and selectivity towards FABP4 as compared to other FABP isoforms. Currently, BMS309403, a potent and highly selective FABP4 inhibitor, is the most commonly studied FABP4 inhibitor in both in vitro and in vivo models, and is indeed the FABP4 inhibitor previously shown to reduce LPS-mediated neuroinflammation in microglia (Kagawa et al. 2023). BMS309403 has an inhibitory constant (K_i_) for FABP4 of less than 2 nM (Floresta et al. 2017), and it has been shown to be very selective for FABP4 over other FABP isoforms (Sulsky et al. 2007). However, despite being highly selective for FABP4, BMS309403 has a very high LogP of 7.2 and a molecular weight of 474.5 Da (PubChem 2021), properties which may reduce the ability of this compound to be formulated as an oral product and to also permeate the blood–brain barrier (BBB). Exhibiting appropriate physicochemical properties to cross the BBB would be critical for any potential therapeutic to access the microglia following peripheral administration, which include a log D in the range of 1–3, a molecular weight < 450 Da and a topological polar surface area (tPSA) of < 60–90Å^2^ (Nicolazzo et al. 2006). While BMS309403 has an appropriate tPSA for BBB penetration (i.e. 64.4 Å^2^), its large molecular weight and high lipophilicity may prevent its ability to traverse the BBB by passive diffusion. Other FABP4 inhibitors have been designed, such as HTS01037, which has high affinity and selectivity for FABP4 and therefore, attenuation of inflammatory mediators from macrophages (Hertzel et al. 2009), however, the polarity of this compound (with a tPSA of 149 Å^2^) (PubChem 2021) would likely limit BBB penetration.

There is therefore a need to develop compounds that are not only able to bind potently and selectively to FABP4, but also exhibit physicochemical properties for optimum BBB penetration so as to more efficiently alleviate microglial-mediated neuroinflammation. Furthermore, these FABP4 inhibitors should exhibit appropriate solubility so that they can be formulated for in vivo application. The overall aim of this study, therefore, was to screen novel FABP4 inhibitors with more appropriate physicochemical properties for BBB penetration, and to evaluate the effectiveness of these compounds in reducing proinflammatory molecule release from LPS-activated microglia. This study assessed the aqueous solubility of four novel FABP4 inhibitors from the same chemical series (i.e. all with a sulfonamide scaffold) as compared to BMS309403, their tolerance in BV-2 cells and their ability to reduce the release of proinflammatory molecules from LPS-activated BV-2 cells, so as to identify a novel FABP4 inhibitor for further preclinical characterisation in neuroinflammation.

## Materials and Methods

### Materials

Dr. Linda J. Van Eldik generously provided the BV-2 cells, which originated from the laboratory of Dr. Elisabetta Blasi (Lexington, KY) (Blasi et al. 1990). Dulbecco’s sterile phosphate buffer saline (PBS), fetal bovine serum (FBS), Hank’s Balanced Salt Solution (HBSS), Pierce BCA Protein Assay kit, and mouse TNF-α uncoated ELISA kit were all purchased from Thermo Fisher Scientific (Grand Island, NY). Dulbecco’s Modified Eagle’s Medium F-12 Ham (DMEM/F-12), trypsin, trypan blue, β-mercaptoethanol, dimethyl sulfoxide (DMSO), d_6_-DMSO, bovine serum albumin (BSA), thiazolyl blue tetrazolium bromide (MTT), penicillin/streptomycin, LPS from Escherichia coli O111:B4, sodium phosphate (Na_2_HPO_4_), sodium chloride (NaCl), 2,2-dimethyl-2-silapentane-5-sulfonic acid (DSS), and 2′,7′-dichlorofluorescin diacetate (DCF-DA) were all purchased from Sigma-Aldrich (St Louis, MO). iTaq Universal Probes One-Step kit (containing 2 × probes RT-PCR reaction mix, iScript reverse transcriptase, and nuclease-free water) was purchased from BioRad (Hercules, CA). QIAshredder columns, RNeasy Mini Plus kit, HiPerfect transfection reagent, FlexiTube FABP4 siRNA (SI02695322), and AllStar Negative Control siRNA were purchased from Qiagen (Hilden, Germany). Taqman gene assays for TNF-α, β-actin, and GAPDH were purchased from Applied Biosystems (Foster City, CA). BMS309403 was purchased from Cayman Chemicals (Ann Arbor, MI). MFP-0012328, MFP-0012314, and MFP-0012318 were purchased from Enamine (Kyiv, Ukraine), while MFP-0011462 was purchased from ChemBridge (San Diego, CA).

### Selection of Compounds Targeting FABP4 and Assessment of their Affinity to FABP4

A prior fragment screen identified a series of bi-aryl sulfonamides that bound to FABP4. Analogues of this chemical sulfonamide scaffold were then purchased, which led to the identification of the compounds used in this study (MFP-0011462, MFP-0012314, MFP-0012318, and MFP-0012328). Their predicted LogP and tPSA values were calculated in Vortex v2020.1.117658-s (Dotmatics, Boston, MA). The binding affinity of these four compounds was characterised by isothermal titration calorimetry (ITC). ITC was carried out using an iTC200 microcalorimeter (MicroCal, United Kingdom) with a coin shaped sample cell (201.4 μL) with stirring at 1000 rpm at 25°C. Titrations were performed in an ITC buffer (50 mM sodium phosphate, 50 mM NaCl, pH 7.4 with 2% DMSO). Compounds were diluted from concentrated stocks in ITC buffer and loaded into the titration syringe at a concentration of 1 mM, with a final DMSO concentration of 2% (v/v). Recombinantly-expressed human FABP4 was prepared as described previously (Lee et al. 2015a) in an identical buffer at a protein concentration of 80 μM and placed in the sample cell. A thermal equilibration step at 25°C was followed by an initial 180 s delay step and subsequently an initial 0.2 μL injection. 16 serial injections of the 1 mM compound solution (2 μL each) were made at intervals of 220 s with continuous stirring of the solution in the sample cell. The iTC200 control software was used to operate and acquire raw data as power (microcalories/sec) vs time (min). The data were processed using the Origin 7.0 software (MicroCal) and the binding affinity (K_D_) was calculated by fitting the data to a one-site binding model.

### 1D ^1^H NMR to Assess Aqueous Solubility

1D ^1^H NMR was employed to determine the solubility limits of the four MFP compounds and BMS309403 in phosphate buffer. This method has been commonly used to determine compound solubility in the drug discovery setting (Devine et al. 2014; Lee et al. 2015b; Lin et al. 2009). A 100 mM of stock solution of each compound was first prepared in d_6_-DMSO. These stock solutions were then added into phosphate buffer (50 mM sodium phosphate, 25 mM NaCl, 100 µM of DSS, pH 7.4 in D_2_O) to achieve a final d_6_-DMSO concentration of 0.1% (v/v) which is equivalent to the DMSO concentration present in BV-2 cell culture experiments. All samples were inspected to note whether visible precipitates were formed. ^1^H NMR spectra for all of the samples were acquired using Bruker Avance III 600 MHz spectrometer at 25 °C. Experimental data were acquired using a relaxation delay of 5 s with 128 scans, and water suppression using an excitation sculpting pulse scheme. Mestrelab Research 13.0 software was used to integrate the proton resonances of the compounds. A well-resolved peak (i.e. no overlaps with any other peaks) in the spectrum of each compound was chosen for integration, relative to the DSS peak (at 0 ppm). The integrated value was then plotted against the compound concentration to determine whether the aqueous solubility was linear over the concentration range tested. In addition, the chemical shifts of the resonances in the 1D ^1^H NMR spectra were monitored. Compounds that showed a linear increase in peak intensity with concentration and no measurable change in chemical shift were considered to be soluble and free from aggregation over the tested concentration range.

### Culturing and Treatment of BV-2 Cells

BV-2 cells were cultured in DMEM/F-12 media supplemented with 10% (v/v) FBS and 1% (v/v) penicillin/streptomycin, and kept at 37 °C in a humidified incubator (95% O_2_, 5% CO_2_) for all studies. Cells with 80% confluency were used for experiments. At 24 h post-seeding, BV-2 cells were treated with either of the MFP compounds (at concentrations detailed below) or vehicle (0.1% (v/v) DMSO) and 1 µg/mL LPS for a period of 24 h. In some experiments (detailed in Results), BV-2 cells were exposed to MFP compounds for 2 h before a 24 h exposure to LPS and MFP compounds, and in some studies, in the presence of FABP4 siRNA using a 24 h treatment that we have previously employed (Low et al. 2021). In brief, BV-2 cells were washed once 4 h post-seeding with blank DMEM/F-12 media. siRNA complexes (containing FABP4 siRNA, target sequence 5’-CTGGATGGAAATTTGCATCAA-3’ or AllStar Negative Control siRNA, target sequence proprietary) were added dropwise onto the cells. siRNA complexes were prepared by mixing siRNA and HiPerfect transfection reagent together in FBS-free DMEM/F12 media and incubating for 15 min to allow the siRNA complexes to form. The cells were incubated with the siRNA complex for 5 min, after which, DMEM/F12 media containing FBS was added into each well to dilute the siRNA complexes to 5 nM.

### MTT Assay to Assess BV-2 cell Viability Following MFP Compound Treatments

To determine the most appropriate and tolerable concentration of MFP compounds in BV-2 cells, an MTT assay was performed. Following treatment with increasing concentrations of MFP compounds for 24 h, BV-2 cells were rinsed once with PBS prior to being incubated with 0.45 mg/mL MTT reagent for 4 h in a 37 °C, 5% CO_2_ incubator. After that, the MTT reagent was removed and 150 µL of DMSO was added into all wells for 30 min. The plate was then immediately read on a Perkin-Elmer Enspire fluorescence plate reader (Boston, MA) at 540 nm. Background subtraction was performed before the percentage cell viability was calculated and expressed as a ratio of mean absorbance of MFP-treated cells over mean absorbance of vehicle-treated cells.

### Measurement of Intracellular ROS

Intracellular ROS in BV-2 cells, following treatment with MFP compounds and LPS, was detected using the widely-used membrane permeable probe, DCF-DA. Post treatment, BV-2 cells were rinsed once with warm HBSS prior to being incubated with 40 µM of DCF-DA for 30 min at 37°C, 5% CO_2_. The wells were then rinsed again with HBSS before being read on a Perkin-Elmer Enspire plate reader (λ_ex_: 488 nm, λ_em_: 535 nm) as we have reported previously (Kagawa et al. 2023).

### Measurement of Extracellular Nitrite

For measurement of extracellular nitrite concentrations, the Griess assay was performed as per the manufacturer’s protocol (Molecular Probes, Eugene, OR) with minor changes. Griess reagent components were separated and added sequentially, with sulfanilic acid added to samples first followed by a 15 min incubation and then *N-*(1-naphthyl)ethylenediamine dihydrochloride was added followed by a further 15 min incubation. Deionised water was not included given standards were prepared in media for comparison with media samples. The absorbance of the media samples was measured on a Perkin-Elmer Enspire plate reader at 548 nm, with nitrite concentrations in media samples obtained by back calculating sample absorbance values back to a standard curve prepared through serial dilution of NaNO_2_ prepared in media.

### Quantification of TNF-α Concentration Using Enzyme-Linked immunosorbent Assay (ELISA)

ELISA was performed as per manufacturer’s protocol to measure the concentration of TNF-α in the supernatant in which BV-2 cells were treated. In short, 100 µL of supernatant, alongside 100 µL of TNF-α standards, were aliquoted into pre-coated ELISA plates. Following a 2 h incubation, the wells were washed with wash buffer (0.05% (w/v) Tween-20 in PBS) four times before 100 µL of detection antibody was added for 1 h. After washing the wells four times, 100 µL of streptavidin-HRP was added for 30 min. The wells were again washed six times before 100 µL of TMB substrate was added for 15 min. 100 µL of Stop solution was added into each well before the plate was immediately read on a Perkin-Elmer Enspire fluorescence plate reader at 450 nm (excitation) and 570 nm (absorbance). Absorbance values at 570 nm were subtracted from 450 nm to account for background. TNF-α concentration was normalised to total protein count (mg), as quantified by a BCA assay, and reported as pg/mg protein.

### RT-qPCR for TNF-α mRNA Expression

RT-qPCR was performed to determine the amount of TNF-α mRNA following the required cell treatments, as previously described (Low et al. 2020). In brief, BV-2 cells were washed twice with ice-cold PBS before RNA isolation was performed using the RNeasy Plus Mini kit as per the manufacturer’s protocol. The concentration and purity of the isolated RNA was assessed using a Thermo Fisher Nanodrop 1000 spectrophotometer (Rockford, IL). RT-qPCR reactions were performed using a BioRad C1000 thermocycler in a CFX96 system (Hercules, CA). In accordance with the manufacturer’s instructions, each PCR reaction mix contained 12.5 μL of 2 × probes RT-PCR reaction mix, 0.5 μL of iScript reverse transcriptase, 6.3 μL of nuclease-free water, 0.695 μL of Taqman primer, and 100 ng of isolated RNA. Thermocycling was performed at 50 °C for 10 min, and then 95 °C for 5 min, followed by 45 cycles of 95 °C for 15 s and 60 °C for 30 s. The threshold cycles (C_t_) were automatically calculated using the CFX manager software. The fold-change method (2^−ΔΔC^_t_), as detailed in the equation below, was used to determine the mRNA levels of TNF-α relative to β-actin and GAPDH (housekeeping genes), and fold changes were calculated as a ratio to vehicle-control cells.

$$\Delta Ct= {C}_{t(TNF-\alpha )}- {(C}_{t\left(\beta -actin\right)}+ {C}_{t\left(GAPDH\right)})/2$$

$$\Delta \Delta Ct= {C}_{t(treated)}- {C}_{t\left(control\right)}$$

$${2}^{-\Delta \Delta Ct}= fold\;change\;of\;TNF-\alpha \;normalised \;to \;\beta -actin \;and \;GAPDH$$

### Data and Statistical Analyses

Data analyses were performed using Graphpad Prism 8 (GraphPad, La Jolla, CA). All data were expressed as mean ± SEM, where all replicates performed were biological replicates. When comparing between two groups, a Student’s unpaired t-test was performed. When more than two groups were compared, an analysis of variance (ANOVA), followed by an appropriate post-hoc test was performed. Values of p < 0.05 were considered statistically significant.

## Results

### Predicted Physicochemical Properties of MFP Compounds and BMS309403

The physicochemical properties of the compounds of interest (i.e. the four MFP compounds) and BMS309403 were predicted to assist in understanding their aqueous solubility and potential to permeate the BBB. These properties including molecular weight, logP and tPSA are provided in Table 1, alongside the K_D_ values against FABP4, determined by ITC (Figure S1).

**Table 1:**
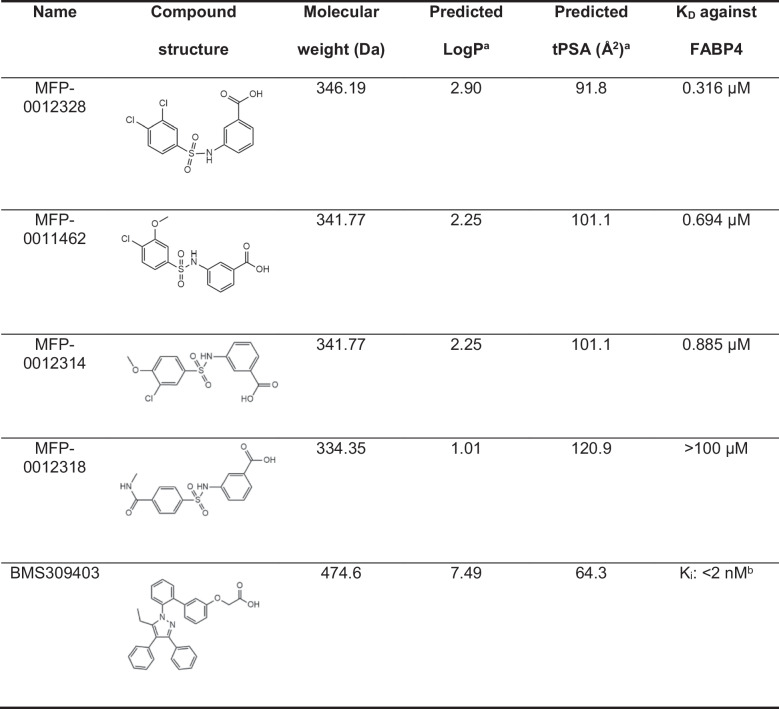
Structures, molecular weight, predicted LogP, predicted tPSA and K_D_ against FABP4 for each of the MFP compounds and BMS309403

^a^Vortex v2020.1.117658-s

^b^This K_i_ was obtained from a previously-published study (Floresta et al. 2017)

**Fig. 1:**
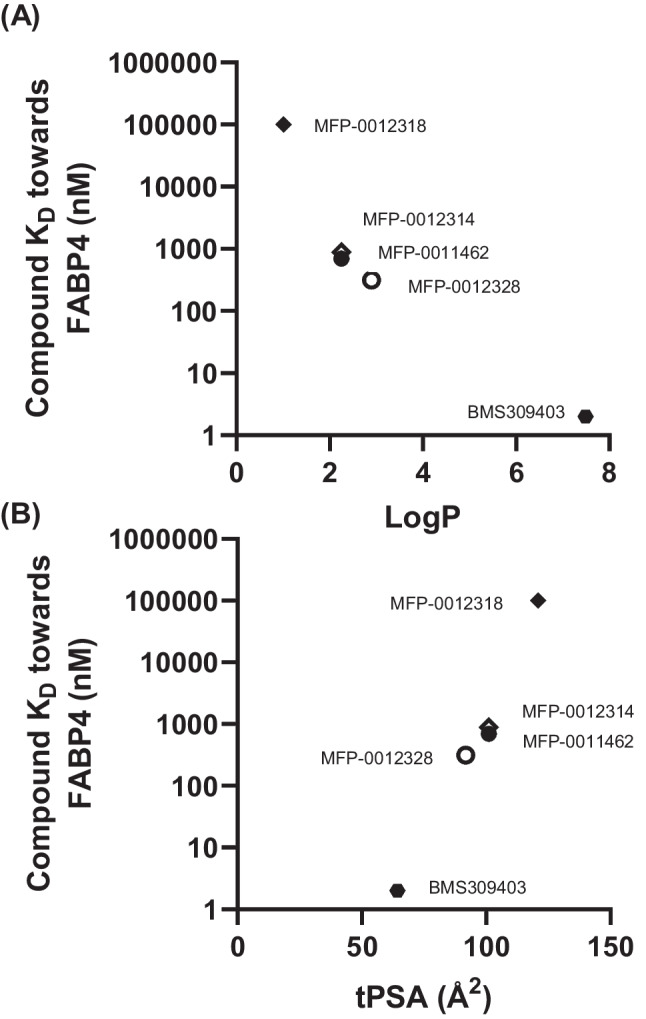
The relationship between K_D_ towards FABP4 and (**A**) LogP and (**B**) tPSA of FABP4 inhibitors. A negative relationship for K_D_ and LogP was observed, while K_D_ and tPSA demonstrated a positive relationship

Since both LogP and tPSA can affect how compounds behave in an aqueous buffer and therefore affect their binding affinity towards FABP4, the relationships between binding affinity, LogP, and tPSA were investigated. From Fig. 1A, a negative relationship for binding affinity and LogP was observed, where compounds with a higher binding affinity towards FABP4 have a higher LogP value. In contrast, Fig. 1B illustrated that there is a positive relationship for binding affinity and tPSA, where compounds with higher binding affinities have lower tPSA values.

### All FABP4 Inhibitors were Soluble in Phosphate Buffer

**Fig. 2:**
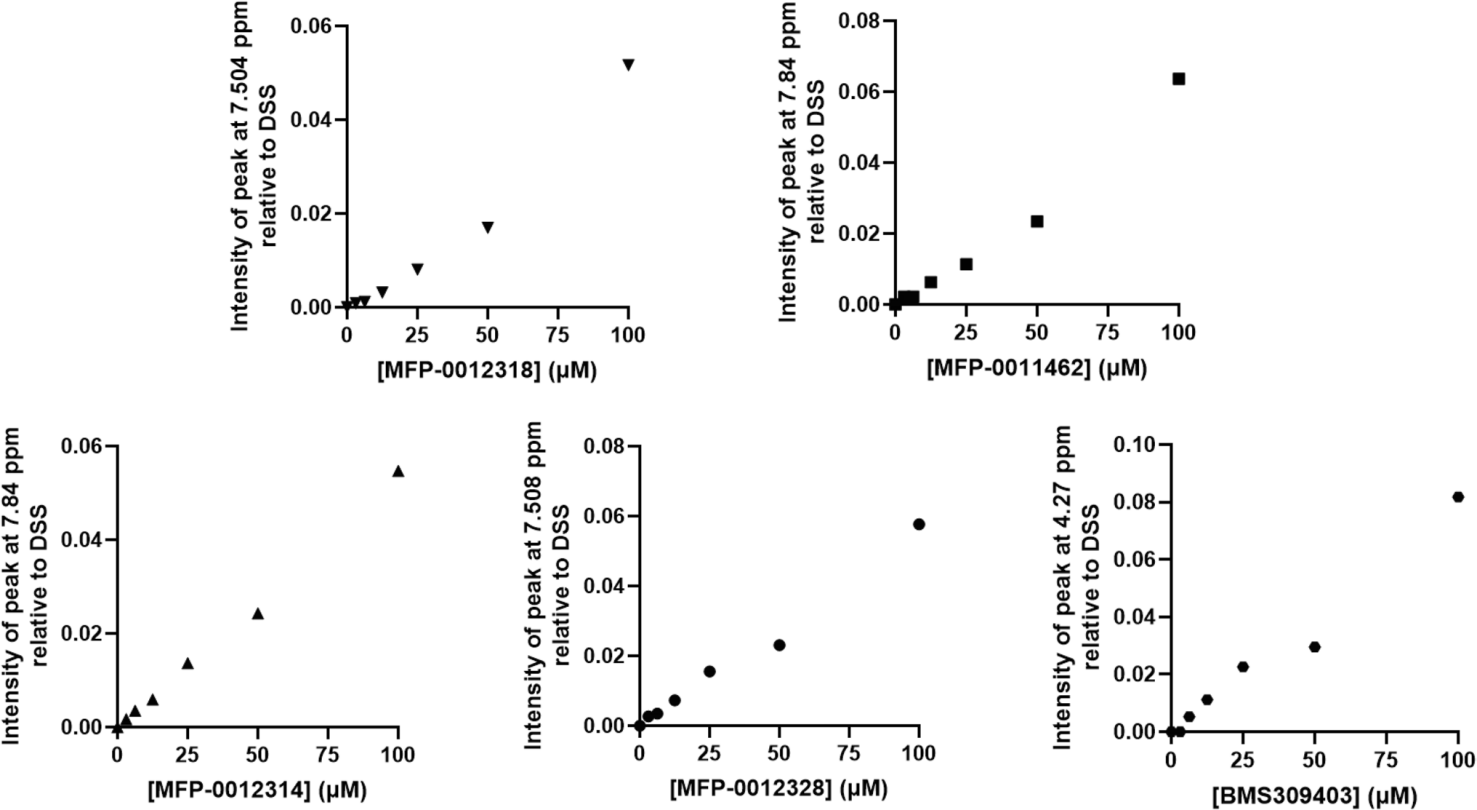
Relative peak intensities of MFP-0012318, MFP-0011462, MFP-0012314, MFP-0012328, and BMS309403 (to DSS) at different concentrations in phosphate buffer, ranging from 3.125–100 µM using ^1^H NMR. Representative ^1^H NMR spectra of each compound at increasing concentration in phosphate buffer are shown in Figure S2

The aqueous solubility of each of the novel FABP4 inhibitors and BMS309403 was assessed using ^1^H NMR. As shown in Figure S2, the signal intensity for all MFP compounds increased proportionally to concentration over the concentration range assessed (Fig. 2), indicating that the compounds were soluble in the phosphate buffer up to 100 µM. There was also no change in chemical shifts with increasing concentrations. Interestingly, the same observation was made for BMS309403, despite its very high LogP value.

Since all MFP compounds showed acceptable solubility in aqueous buffer, these compounds were then screened using in vitro assays to determine any potential toxicity and if their difference in binding affinity to FABP4 correlated to how well they were able to reduce the release of proinflammatory molecules from LPS-activated BV-2 cells.

### MFP Compounds were well Tolerated at Moderate Concentrations in BV-2 Cells

**Fig. 3:**
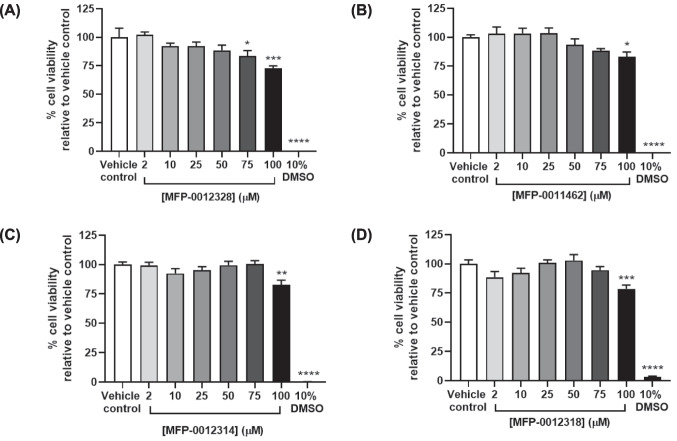
BV-2 cell viability following treatment with increasing concentrations of MFP compounds. Relative to vehicle control, no significant differences in BV-2 cell viability were observed following treatment with 2–50 µM of MFP-0012328, MFP-0011462, MFP-0012314, and MFP-0012318, while treatment with 75 µM and 100 µM of MFP-0012328 and 100 µM of MFP-0011462, MFP-0012314, and MFP-0012318 resulted in a 0.17–0.27-fold reduction in cell viability. 10% DMSO served as a positive control. Data are represented as mean ± SEM (n = 6 biological replicates), where *p < 0.05, **p < 0.01, ***p < 0.001, and ****p < 0.0001, as assessed using a one-way ANOVA, followed by a post-hoc Dunnett’s test

Before assessing the ability of these MFP compounds to reduce proinflammatory mediator release from LPS-activated BV-2 cells, an MTT assay was performed to identify their tolerable concentrations in these cells. As shown in Fig. 3, all MFP compounds were well tolerated up to a concentration of 50 µM. Higher concentrations of all compounds resulted in a 17.0–27.3% reduction in cell viability. Therefore, 50 µM of each compound was used for subsequent studies, which is consistent with the concentration of BMS309403 previously shown to reduce LPS-mediated release of inflammatory mediators from BV-2 cells (Kagawa et al. 2023).

### MFP Compounds Reduced LPS-Mediated ROS Production in BV-2 Cells in a Concentration-Dependent Manner

Concentrations of 50 µM of MFP-0012328, MFP-0011462, MFP-0012314, and MFP-0012318 were all found to significantly reduce ROS production relative to LPS-only treated cells, as summarised in Table 2. This result was unexpected as it suggested that binding affinity of the inhibitor towards FABP4 plays no role in determining inhibitor efficacy (given that MFP-0012318 exhibits a K_D_ for FABP4 exceeding 100 µM). To ensure that the concentration of MFP compounds used was not saturating FABP4 in these studies, a lower concentration of MFP compounds was used. Interestingly, at 10 µM, the reduction in intracellular ROS production was associated with the binding affinity of the compound towards FABP4, as MFP-0012328 (K_D_: 0.316 µM) was able to reduce ROS production the most, while MFP-0012318 had no effect on ROS production. This indicated that binding affinity to FABP4 played a role in the ability to attenuate LPS-mediated ROS production. Given that MFP-0012328 reduced ROS production the most, this compound was further investigated in subsequent studies.

**Table 2:** Intracellular ROS production in BV-2 cells following pre-treatment with 50 µM or 10 µM of MFP compounds for 2 h, followed by co-treatment with MFP compounds and 1 µg/mL LPS for 24 h. **p < 0.01, ***p < 0.001, and ****p < 0.0001 compared to LPS-only treated BV-2 cells, n = 6 biological replicates

Compound	% reduction in intracellular ROS production (mean ± SEM)	
	50 µM	10 µM
MFP-0012328	23 ± 0.10 (**p < 0.01)	51 ± 0.07 (****p < 0.0001)
MFP-0011462	46 ± 0.08 (***p < 0.001)	49 ± 0.04 (****p < 0.0001)
MFP-0012314	42 ± 0.07 (***p < 0.001)	40 ± 0.09 (**p < 0.01)
MFP-0012318	54 ± 0.09 (****p < 0.0001)	0.5 ± 0.10 (ns)

### MFP Compounds Reduced LPS-Mediated Nitrite Release from BV-2 Cells

**Fig. 4:**
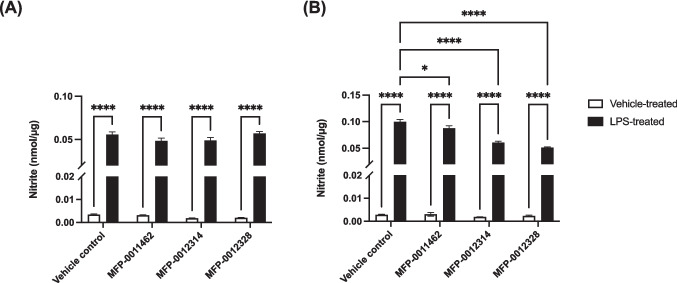
Nitrite release from BV-2 cells following treatment with MFP-0011462, MFP-0012314 or MFP-0012328 at (**a**) 10 µM or (**b**) 50 µM in the presence and absence of 1 µg/mL LPS for 24 h. LPS was able to significantly increase nitrite release from BV-2 cells after 24 h. Treatment with all MFP compounds significantly reduced LPS-mediated nitrite release from BV-2 cells at 50 µM, but not at 10 µM. Data are represented as mean ± SEM (n = 4 biological replicates), where *p < 0.05 and ****p < 0.0001, as assessed using a two-way ANOVA, followed by a post-hoc Tukey’s test

As a secondary readout, the ability of the MFP compounds to attenuate LPS-mediated BV-2 release of nitrite was examined. Given that MFP-0012318 did not reduce ROS production at a concentration of 10 µM, this compound was not evaluated for its nitrite-attenuating effects. As expected, LPS significantly increased the release of nitrite from BV-2 cells (Fig. 4). Interestingly, all compounds attenuated the LPS-mediated release of nitrite from BV-2 cells when used at a concentration of 50 µM ranging from 12.2% for MFP-0011462 to 51.3% for MFP-0012328 (Fig. 4B), however, these compounds elicited no effect on LPS-mediated nitrite release when used at a concentration of 10 µM, suggesting that ROS production is more sensitive to these MFP compounds than is nitrite release from the BV-2 cells.

### The ROS-Attenuating Effects of MFP-0012328 were FABP4 Mediated

**Fig. 5:**
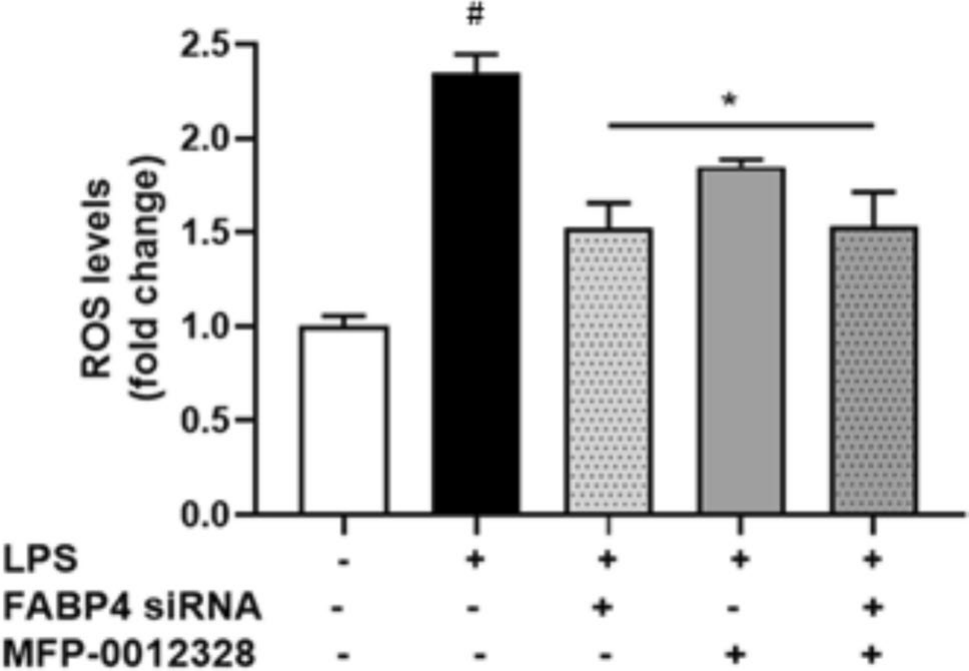
ROS production in BV-2 cells following treatment with either 5 nM of FABP4 siRNA, 10 µM MFP-0012328, or both, in the presence of 1 µg/mL LPS for 24 h. LPS was able to significantly increase ROS production in BV-2 cells after 24 h. Treatment with either FABP4 siRNA, MFP-0012328, or with both, significantly reduced ROS production by 36.6–61.2%, as compared to the LPS-treated BV-2 cells. Data are represented as mean ± SEM (n = 6 biological replicates), where *p < 0.05 when compared to LPS-only treatment and #p < 0.05 when compared to control treatment, as assessed using a one-way ANOVA, followed by a post-hoc Tukey’s test

To ensure that the effect of MFP-0012328, as the most effective ROS-attenuating compound, was mediated through inhibition of FABP4, and not on other targets, the effect of MFP-0012328 was assessed in the presence and absence of FABP4 siRNA. From Fig. 5, it can be observed that MFP-0012328 was not able to reduce ROS levels any further in FABP4 siRNA treated cells relative to BV-2 cells with FABP4 present. This indicated that the ROS-reducing effects of MFP-0012328 were most likely mediated through inhibition of FABP4.

### MFP-0012328 Reduced TNF-α mRNA Expression and Protein Concentration in LPS-Activated BV-2 Cells

**Fig. 6:**
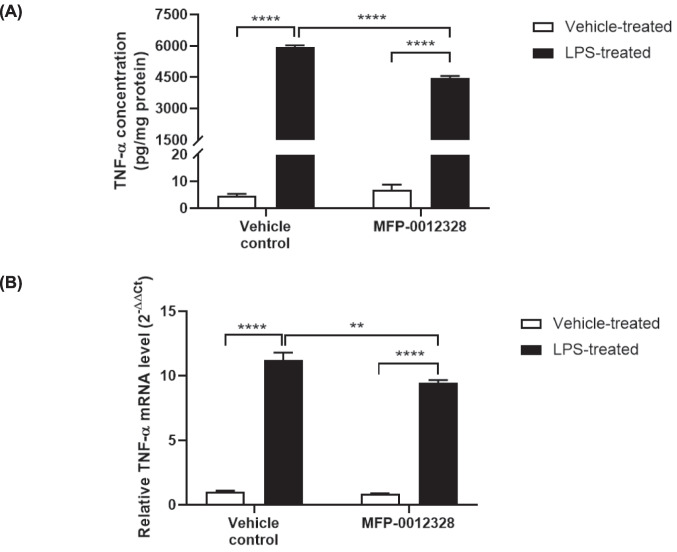
(**A**) The amount of TNF-α protein released by BV-2 cells following treatment with 10 µM of MFP-0012328 and 1 µg/mL of LPS for 24 h. TNF-α concentration in LPS-treated BV-2 cells was reduced 24.8% by MFP-0012328 relative to LPS alone-treated BV-2 cells. Data are represented as mean ± SEM (n = 6 biological replicates), where ****p < 0.0001, as assessed using a two-way ANOVA, followed by a post-hoc Tukey’s test. (**B**) mRNA of TNF-α in BV-2 cells following treatment with 10 µM of MFP-0012328 and 1 µg/mL of LPS for 24 h. TNF-α mRNA levels in LPS-treated BV-2 cells were reduced 17% by MFP-0012328 relative to LPS alone-treated BV-2 cells. Data are represented as mean ± SEM (n = 4 biological replicates), where **p < 0.01 and ****p < 0.0001, as assessed using a two-way ANOVA, followed by a post-hoc Tukey’s test

We further investigated if MFP-0012328 was also able to reduce the concentration of the common proinflammatory molecule, TNF-α, in addition to its ROS and nitrite attenuating effects. As shown in Fig. 6A, pre-treating BV-2 cells with MFP-0012328 led to a significant (24.8%) reduction in extracellular concentration of TNF-α relative to LPS-only treated cells. The reduction in extracellular TNF-α concentration correlated to a reduction in TNF-α mRNA production (Fig. 6B), confirming that the effect of MFP-0012328 on TNF-α was transcriptional in nature. These results suggested that MFP-0012328 is a useful FABP4 inhibitor in reducing three inflammatory mediators from activated microglia.

### MFP-0012328 Reduced ROS Production and TNF-α Release from BV-2 Cells post LPS Activation

**Fig. 7:**
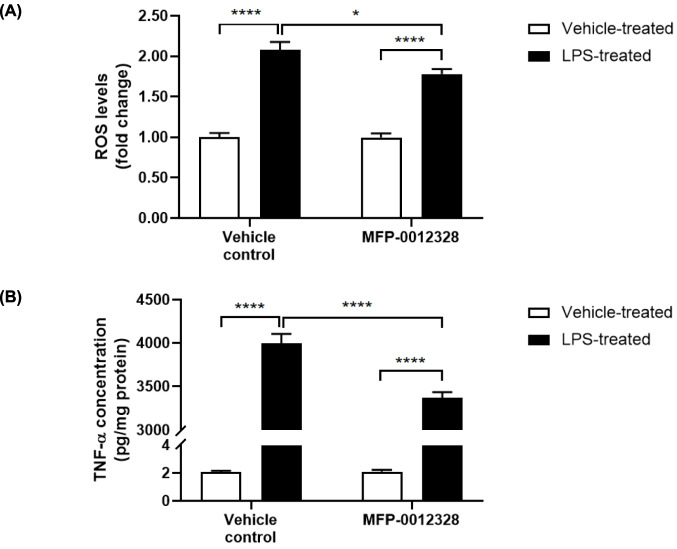
(**A**) ROS production and (**B**) amount of TNF-α protein released from BV-2 cells that had been pre-treated with LPS for 4 h, followed by the addition of 10 µM of MFP-0012328. ROS levels and TNF-α release were reduced by 27.8% and 15.7%, respectively, in the presence of LPS, as compared to LPS-alone treated BV-2 cells. Data are represented as mean ± SEM (n = 6 biological replicates), where *p < 0.05 and ****p < 0.0001, as assessed using a two-way ANOVA, followed by a post-hoc Tukey’s test

While we have demonstrated that pre-treating BV-2 cells with MFP-0012328 was able to reduce ROS production and TNF-α concentration when administered prior to, or in combination with LPS, it was important to confirm that MFP-0012328 still attenuated the release of these proinflammatory molecules following acute LPS activation. Even when BV-2 cells were pre-treated with LPS for 4 h prior to MFP-0012328 exposure, MFP-0012328 was still able to significantly reduce both ROS production and TNF-α concentration, by 27.8% and 15.7%, respectively (Fig. 7), albeit the reduction was not as high as when BV-2 cells were treated with the inhibitor before, or in combination with, exposure to LPS.

## Discussion

The overactivation of microglia, which leads to the excessive release of proinflammatory molecules, is the main contributor to neuroinflammation. Therefore, efforts to modulate the proinflammatory microglial phenotype can lead to new therapeutic options to reduce neuroinflammation in diseases such as AD, PD and MND. Specific and selective FABP4 inhibitors have been developed which may attenuate microglial-mediated neuroinflammation, given that FABP4 is implicated in this process (Kagawa et al. 2023; Duffy et al. 2017), however their physicochemical properties may hinder their development as potential therapeutic entities. This study therefore investigated the potential of novel FABP4 inhibitors, identified using fragment screening and with more drug-like properties, to reduce LPS-mediated inflammation in BV-2 cells since these cells express similar phenotypical, phagocytic, and secretive functions as primary microglia (Henn et al. 2009).

In order to assess the aqueous solubility of each of the compounds,^1^H-NMR studies were performed in phosphate buffer, which is a widely considered and representative buffer consistent with those used in in vitro pharmacology assays (LaPlante et al. 2013a, 2013b). It is important for therapeutic compounds to exhibit a high enough aqueous solubility to ensure that the compound can be easily formulated into a dosage form and for subsequent bioavailability following oral administration (Box and Comer 2008; Lipinski et al. 2001; Williams et al. 2013). ^1^H NMR studies showed that all MFP compounds with the sulfonamide scaffold were soluble up to 100 µM in phosphate buffer. Interestingly, despite the high LogP of BMS309403 (PubChem 2021), it also remained in solution at concentrations of up to 100 µM in phosphate buffer. This unexpectedly high solubility may have been due to the small amount of d_6_-DMSO present in the phosphate buffer, which resulted in the apparent solubility of BMS309403 being largely kinetically driven. Compounds delivered in a DMSO solution to an aqueous medium are usually in a very high energy state, which could enhance apparent solubility (Lipinski et al. 2001). Therefore, in order to fully quantify the aqueous solubility of the MFP compounds and also BMS309403, equilibrium solubility studies would be required. Since all MFP compounds tested were soluble in aqueous buffer (containing a small amount of d_6_-DMSO) up to a concentration of 100 µM, this suggests that they may exhibit suitable solubility for oral administration, albeit this would require confirmation through in vivo oral pharmacokinetic studies.

As the MFP compounds in this study have not been tested in any in vitro assays, a cell viability study was first performed to ensure that the concentration chosen for all compounds was well tolerated in BV-2 cells. This is critical to ensure that any changes observed in subsequent pharmacological assays are a specific result of the inhibitor, and not generalised cell death. Given that we were interested in assessing the greatest effect that these compounds would have on proinflammatory molecule release from LPS-activated microglia, the highest tolerable concentration of each of the different inhibitors was initially chosen (i.e. 50 µM), which is a concentration of BMS309403 that we have previously demonstrated also does not exhibit toxicity to BV-2 cells (Kagawa et al. 2023). Although the inhibitor concentrations used in this study were higher than their reported K_D_ values, this is common practice for inhibitor studies (Hui et al. 2010; Furuhashi et al. 2007; Suhre et al. 2011). These higher concentrations of inhibitors were employed given the possibility of non-specific adsorption onto cell culture plasticware reducing the free concentration available to exert an effect and the likely differences in cellular access of each inhibitor. By utilising a concentration substantially higher than the K_D_, we can be more confident that the concentration of each inhibitor present at the target site (i.e. cytoplasmic FABP4) would be above the inhibitor K_D_. This concentration of 50 µM resulted in all inhibitors significantly reducing intracellular ROS production, regardless of their binding affinity towards FABP4, which was unexpected. This phenomenon could be due to an excessive amount of inhibitor saturating all targets within microglia, thus potentially resulting in off-target effects. For this reason, the effect of each of the inhibitors on pro-inflammatory mediator release was assessed at a concentration of 10 µM. Compounds with a higher K_D_ value towards FABP4 (but lower LogP value) were not able to reduce ROS production in LPS-activated microglia as effectively as those with the lower FABP4 K_D_ value (Table 2), suggesting that binding affinity of MFP compound to FABP4 does correlate to ability to inhibit the production of ROS, but not the release of nitrite, from BV-2 cells. While this relationship is expected, it is also theoretically possible that the difference in ability to attenuate inflammatory mediator release is associated with different BV-2 cellular uptake of each inhibitor, and therefore, BV-2 cellular uptake studies could be undertaken to ascertain this possibility. Ultimately, assessment of BBB penetration and microglial uptake following peripheral administration of these FABP4 inhibitors in vivo would support the appropriateness of the concentrations of these compounds used in these in vitro studies. Importantly, the MFP compounds exhibited similar anti-inflammatory effects as we have previously reported for BMS309403 in BV-2 cells. For comparison, a 50 µM concentration of MFP compounds led to a 23.0–54.0% reduction in ROS levels (similar to a 36.0% reduction mediated by the same concentration of BMS309403 (Kagawa et al. 2023)) and a 12.2–51.3% reduction in nitrite production (similar to a 26.8% reduction mediated by 50 µM of BMS309403) (Duffy et al. 2017). Interestingly, MFP-0012328 at a concentration of 10 µM led to a 24.8% reduction in LPS-mediated TNF-α release from BV-2 cells, which was similar to the effect of BMS309403 at a five-fold higher concentration (i.e. 29.0%) (Kagawa et al. 2023). This suggests a more potent effect of MFP-0012328 which may be attributed to greater BV-2 cellular access, given that MFP-0012328 has a substantially higher K_D_ than BMS309403.

Since MFP-0012328 was able to reduce LPS-induced ROS production most significantly, this compound was further investigated in a proinflammatory cellular setting. Further studies demonstrated that MFP-0012328 was also able to reduce the release of ROS and TNF-α following acute LPS stimulation (Fig. 7), implying its ability to modulate the proinflammatory microglial phenotype even after the onset of inflammation, which is more clinically relevant. Furthermore, we have also demonstrated that BV-2 cells treated with MFP-0012328 and FABP4 siRNA (which targets the FABP4 mRNA sequence directly) resulted in a similar fold change in ROS production in the presence of FABP4, indicating that the effect of MFP-0012328 on ROS production was FABP4-specific, and not due to any off-target effects, as we have previously demonstrated for BMS309403 (Kagawa et al. 2023). We have previously demonstrated that treatment of BV-2 cells with FABP4 siRNA results in a 78.0–92.3% reduction in FABP4 mRNA and 74.5–81.7% reduction in FABP4 protein (Low et al. 2021), supporting a critical role of FABP4 in the anti-inflammatory effects of MFP-0012328. These studies therefore suggest that MFP-0012328 could be explored as a potential therapeutic tool to reduce FABP4-mediated neuroinflammation in diseases where microglial FABP4 is upregulated.

Despite the promising results reported in this study, the accessibility of MFP-0012328 and other MFP compounds to microglial FABP4 in an in vivo setting, following for example, oral administration, remains to be elucidated. Furthermore, whether the more favourable physicochemical properties of these compounds, relative to the more potent BMS309403, leads to improved CNS exposure requires evaluation. Therefore, further studies should be undertaken to assess the ability of these compounds to permeate the BBB using relevant approaches, firstly through in an in vitro setting through the use of primary brain microvascular endothelial cells and furthermore, following in vivo administration to rodents (Nicolazzo et al. 2006). This could be assisted through the use of radiolabelled analogues of these compounds, as has been possible with the synthesis of ^14^C-BMS309403 (Okada et al. 2012) or through LCMS/MS quantification of unlabelled compounds (Kooijmans et al. 2012). Furthermore, these compounds should also be tested for their efficacy, especially in an in vivo model of inflammation, to ensure that they exhibit similar effects in more complex systems. In addition, due to sex differences potentially impacting on the degree of neuroinflammation (Osborne et al. 2018), assessing whether these novel FABP4 inhibitor analogues affect proinflammatory molecule release from microglial in a sex-dependent manner is of interest.

## Conclusion

Overall, this study demonstrated that a series of novel FABP4 inhibitors with physicochemical properties more suitable for BBB penetration effectively reduce the release of proinflammatory molecules from LPS-activated microglia. Despite both MFP-0012328 and BMS309403 being soluble in phosphate buffer up to 100 µM, the substantially lower LogP of MFP-0012328 would likely make it an easier drug candidate to scale up and formulate, and hence a more likely candidate to move forward in the drug development process. Therefore, further studies such as drug characterisation, pharmacokinetic/pharmacodynamic studies, and efficacy studies should be performed to support the proposal that MFP-0012328 could indeed be a potential therapeutic compound that may be used to alleviate microglia-induced neuroinflammation in many neurodegenerative diseases.

## Supplementary Information

Below is the link to the electronic supplementary material.

Supplementary file1 (DOCX 1040 KB)

## Data Availability

No datasets were generated or analysed during the current study.
