# Supplementary material for: Evaluation of the Anti-Inflammatory Effects of Novel Fatty Acid-Binding Protein 4 Inhibitors in Microglia
Source: J Neuroimmune Pharmacol. 2025 Apr 16;20(1):40. doi: 10.1007/s11481-025-10191-9 (PMC12000251; doi:10.1007/s11481-025-10191-9)
Supplement: Supplementary file 1 — Supplementary file1 (DOCX 1040 KB) [file 11481_2025_10191_MOESM1_ESM.docx]

**SUPPLEMENTARY INFORMATION**

1. (B)

K_D_ = 694 nM K_D_ = 316 nM


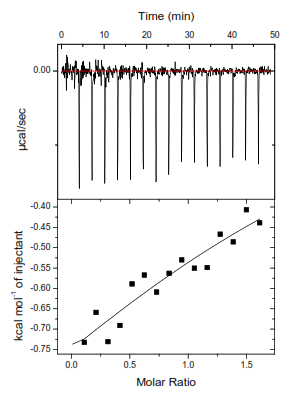

(C) (D)

K_D_ = 885 nM

**Figure S1.** ITC titration of FABP4 with (A) MFP-0011462, (B) MFP-0012328, (C) MFP-0012314 and (D) MFP-0012318. The upper panel shows the raw data from a single ITC experiment and the lower panel shows the corresponding binding isotherm generated from the data fitted to a one-site binding model.

1. (B)


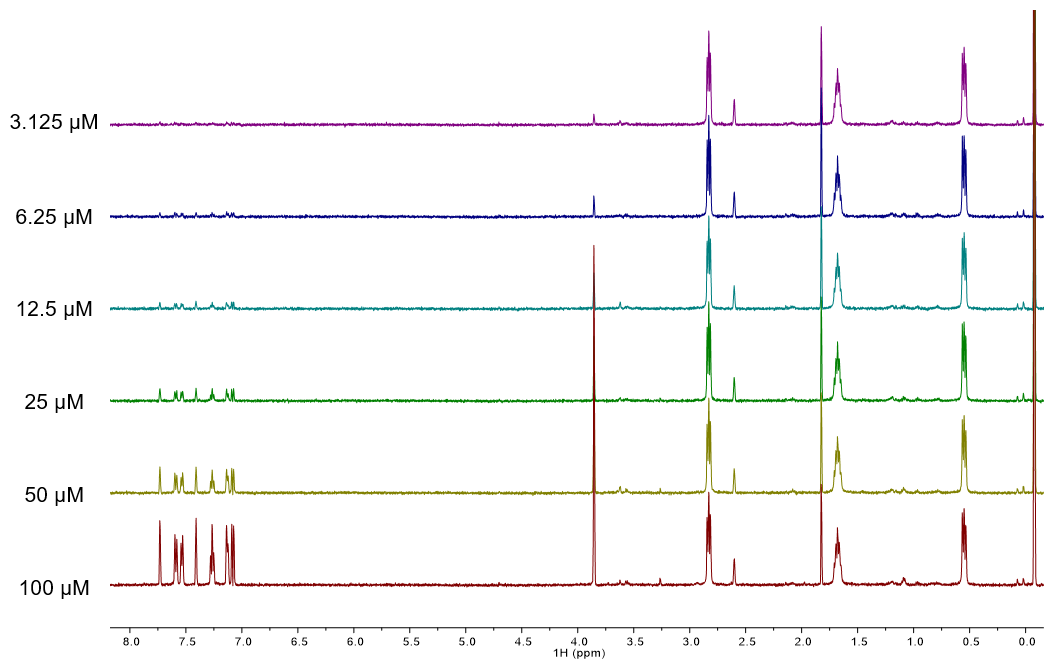


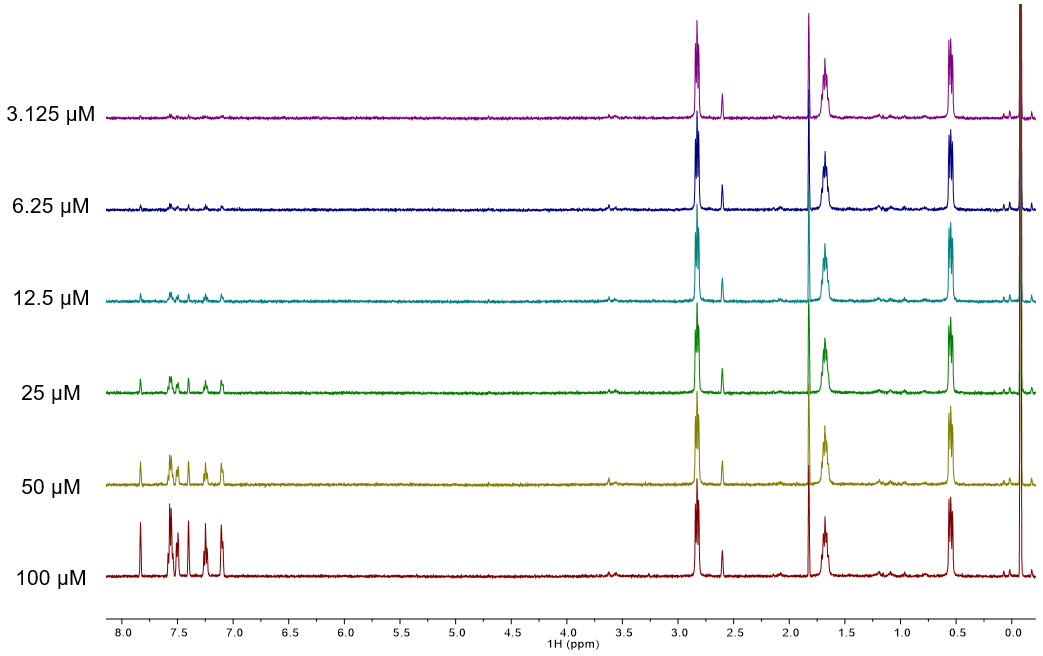


1. (D)


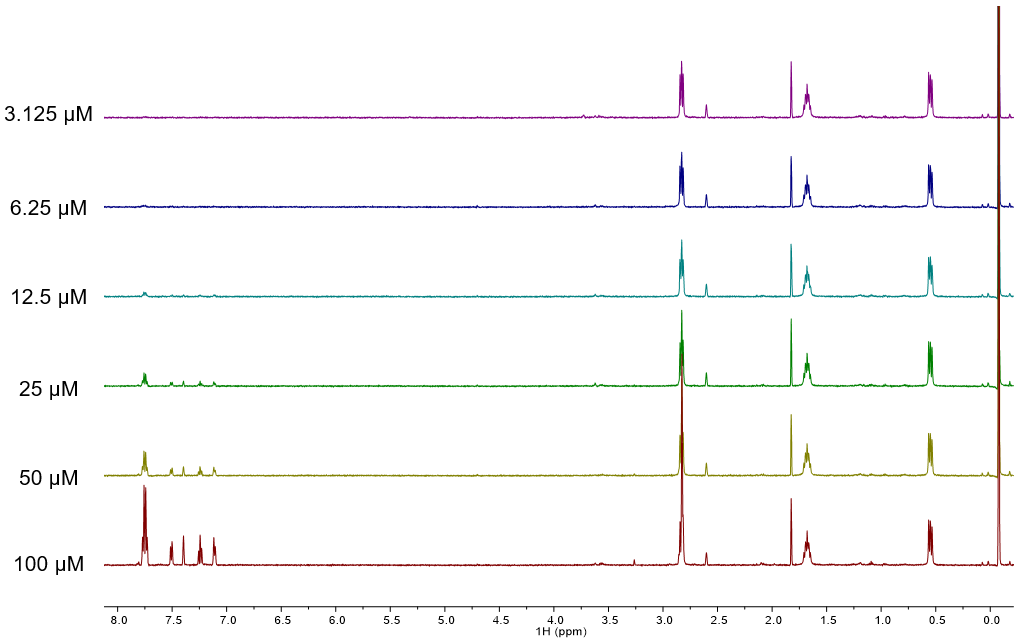


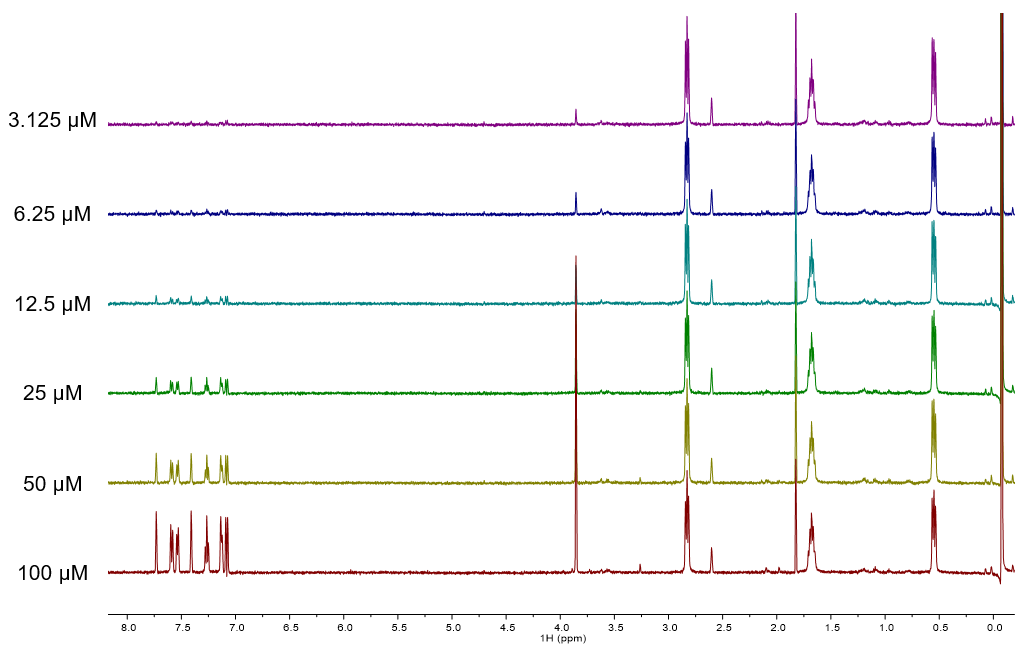


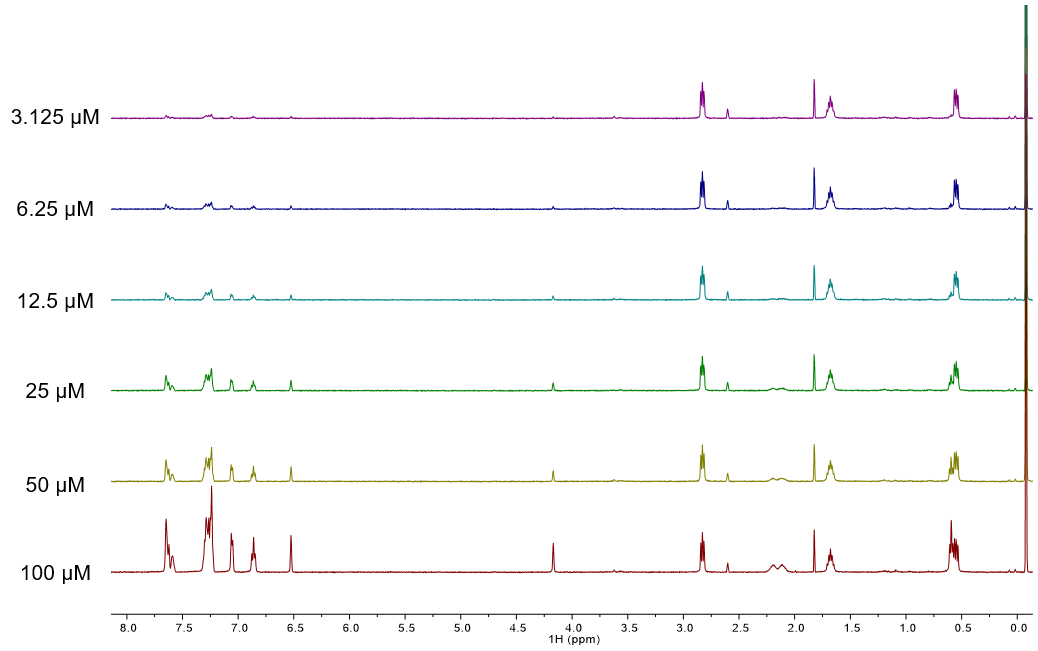


**Figure S2.** ^1^H NMR spectra and concentration dependent chemical shifts of (A) MFP-0012328, (B) MFP-0011462, (C) MFP-0012314, (D) MFP-0012318, and (E) BMS309403 at different concentrations in phosphate buffer, ranging from 3.125-100 µM.
